# The Differentiation Potential of Apical Papilla Cells in Relation to Tenascin-C and Syndecan-1 Expression and Their Potential Role in Regeneration

**DOI:** 10.1155/2024/7295498

**Published:** 2024-09-20

**Authors:** K. Kodonas, A. Fardi, S. Papadimitriou, C. Gogos

**Affiliations:** ^1^ Department of Endodontology School of Dentistry Aristotle University of Thessaloniki, Thessaloniki, Greece; ^2^ Department of Dentoalveolar Surgery Surgical Implantology and Radiology School of Dentistry Aristotle University of Thessaloniki, Thessaloniki, Greece; ^3^ Department of Companion Animal Clinic School of Veterinary Medicine Aristotle University of Thessaloniki, Thessaloniki, Greece

**Keywords:** dental papilla, regenerative endodontics, syndecan-1, tenascin, tooth apex, tooth root

## Abstract

**Introduction:** This study investigated the distribution pattern of tenascin-C and syndecan-1 in the dental mesenchyme during root development of immature swine teeth in order to define the differentiation dynamics of both pulp tissue progenitors and apical papilla cells, as well as to assess the adequacy criticize of the apical papilla to induce dentin–pulp regeneration.

**Methods:** Three 7-month-old miniature swine were used in this study. A total of 12 teeth, including two immature permanent incisors and two premolar teeth of each case, were extracted and processed for histological and immunohistochemical analysis. Different populations of mesenchymal cells located at the root apex were morphologically evaluated in hematoxylin–eosin serial sections. Additionally, the distribution patterns of tenascin-C and syndecan-1 were assessed immunohistochemically.

**Results:** Syndecan-1 was strongly expressed in the dental pulp, particularly along the odontoblasts of the root and the newly deposited predentin layer. Tenascin-C was intensely expressed in the dental pulp. The apical papilla and dental follicle showed no expression of either molecule.

**Conclusions:** Cell differentiation potential in the developing swine apex is progressively restricted to the newly formed dental pulp, whereas phenotypic expression of apical papilla cells remains undetermined unless the new microenvironment triggers cell differentiation towards the odontoblastic lineage.

## 1. Introduction

Today, the existence of tooth specific stem cells has been documented [1–4] and dental stem/progenitor cells are routinely isolated from immature teeth [5, 6]. During regenerative endodontic procedures (REPs) mesenchymal progenitor cell homing is attempted by a massive cell influx in the root canal because of the evoked bleeding. The main source of these cells is thought to be the apically located mesenchymal reservoir of the undifferentiated cells of apical papilla that survive and retain their differentiation potential after endodontic infection [7]. By stimulating apical papilla with a file, the root canal space is filled with blood containing high concentration of mesenchymal progenitors [8]. As the blood coagulates, the newly formed blood clot not only serves as a reservoir of apical papilla progenitor cells but also as a scaffold that promotes cell proliferation and differentiation [9, 10]. However, the histological features, including cellular characteristics and microenvironmental conditions of the open apex have not been extensively studied.

In the developing root, mesenchymal cell populations fully differentiate on a specific time schedule. Considering that the regenerative potential of each cell population is directly linked to the differentiation process, it should be expected that mesenchymal cells of different origin do not have the same dynamics. This fact should not be disregarded during clinical applications of regenerative procedures where traditionally the main source of healing cells is the mesenchymal reservoir of the remaining dental pulp and/or the apical papilla. Currently, there is limited information on the ontogeny, potential, and lineage relationships of mesenchymal progenitor cells residing on the developing root, as well as the role of the local microenvironment in their terminal differentiation. Understanding these mechanisms may be crucial in designing new regeneration protocols. Classical developmental studies and tissue recombination experiments on tooth morphogenesis have shown that cellular differentiation is regulated by epithelial–mesenchymal interactions [11–14]. As the inductive potential shifts from dental epithelium to the mesenchyme, the residing mesenchymal cells are induced into the dental cell lineage [15–17]. These morphogenetic changes are correlated with the distribution pattern of different molecules in the extracellular matrix [18, 19].

Two molecular markers for dental mesenchyme, the extracellular matrix glycoprotein Tenascin and the transmembrane proteoglycan Syndecan, have been detected during tooth crown morphogenesis and differentiation [20]. However, their expression patterns during root development and their association with the commitment process of dental mesenchymal progenitor cells have not been fully understood. Therefore, localizing these molecules using specific antibodies and immunohistology will contribute to revealing the lineage commitment process of mesenchymal progenitor cells in developing root.

The purpose of this study was to investigate the distribution pattern of tenascin-C and syndecan-1 in dental pulp and apical papilla during early root morphogenetic stages to define the differentiation dynamics of pulp tissue and apical papilla progenitors and further understand their role in REPs.

## 2. Material and Methods

All animal experiments were approved by the National Institute of Animal Care (protocol: 13/16956) and the Ethics Committee of Aristotle University of Thessaloniki. All procedures regarding the handling and care of experimental animals complied with European regulations (Directive 2010.63.EU). Two healthy potbellied female and one male 7-month-old minipig weighting from 44 to 58 kg were used, without any specific selection criteria. In dental research, the swine model has been shown to provide proper analogies and resemblances to humans [21, 22]. Consequently, research data can also be indicative of the human situation as well. During the experiments, animal care was conducted in accordance with institution guidelines (EL 54 BIO 18). Animals were housed individually in temperature-controlled rooms and were monitored for activity and feeding by specialized personnel from the Department of Companion Animal Clinic at the School of Veterinary Medicine of Aristotle University of Thessaloniki.

All animal procedures were conducted without compromising the welfare of the animals used. Special care taken to reduce any pain, suffering, or distress during surgery or postoperatively. After sedation with 1 mg/kg xylazine (Alfasan, Woerden, The Netherlands) general anesthesia was induced with an intramuscular injection of 6 mg/kg thiopentone (Hospira, Warwickshire, UK) and maintained using halothane (Concord Pharmaceuticals, Bristol, UK; 1.5%–2.5%) in oxygen, delivered through a breathing circuit. Following surgery, the animals were closely monitored by specially trained staff to detect any signs of distress or pain. In cases where such signs were observed postoperative analgesics (Meloxicam, Metacam 20 mg/mL; Boehringer Ingelheim Ltd., Burlington, ON, Canada at the label dose of 0.4 mg/kg) were administered.

The sample size was calculated using ClinCalc (ClinCalcLLC., Chicago, IL, USA) an online sample calculation software. Based on the outcomes of previous literature [20, 23–26], the alpha error (*α*) was <5% and the test power (1-*β*) was 80%. Therefore, the sample size was set to 12 teeth (six incisors and six premolar teeth). Since tenascin-C and syndecan-1 are known to be expressed in newly differentiated mesenchymal cells, dental pulp cells from the same samples (six incisors and six premolars) were used as positive controls because they express the epitope of interest. Randomization of the sample was not applicable. The intervener as well as the researcher conducting immunohistochemical evaluation were blinded to the experimental units, minimizing potential bias and the effects of confounding factors.

**Figure 1:**
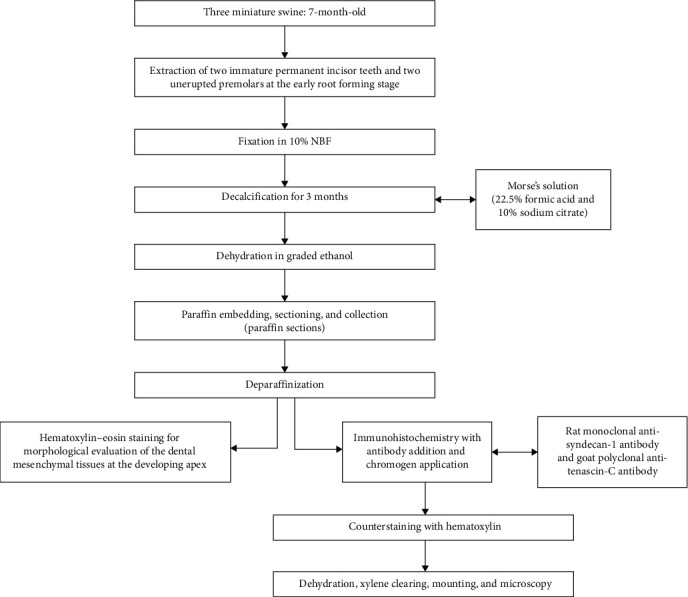
Flow chart of experimental procedures.

Two immature permanent incisor teeth and two unerupted premolars at the early forming stage (Cvek stage II or III) were extracted from each minipig. The teeth were denuded of any soft tissue and fixed in 10% natural buffered formalin in order to be processed for histology and immunohistochemical staining (Figure 1).

After being fixed in 10% natural buffered formalin for 24 h, specimens were then decalcified with Morse's solution (22.5% formic acid and 10% sodium citrate) for 3 months [27]. Demineralized specimens were dehydrated in graded ethanol, cleared in xylene, and embedded in paraffin. Serial sections (5 μm) were cut and placed on positively charged slides (Superfrost Plus Thermo Scientific, Portsmouth, UK). Consecutive tissue sections were obtained for each tooth and evaluated by immunohistology, while one out of every five sections was processed for routine histology using hematoxylin–eosin staining.

Sections on glass slides were deparaffinized in xylene and then substituted with ethanol. A rat monoclonal antibody (antisyndecan-1, product number MAB2780) and a goat polyclonal antibody (anti-tenascin-C, product number AF3358) were commercially obtained (R&D Systems, Minneapolis, USA). The HRP-DAB System (R&D Systems, Minneapolis, USA) was utilized as a staining kit following the manufacturer's instructions. Specifically, antigen detection relied on the formation of the avidin–biotin complex with the primary antibody that interacted with tissue antigens. The chromogenic substance used for visualizing antigen localization on the tissue was 3,3-diaminobenzidine (DAB) which is enzymatically converted by horseradish peroxidase (HRP) into a brown chromogen. Sections were incubated with primary antibodies diluted in PBS (0.2 mg/mL for anti-tenascin-C and 0.5 mg/mL for anti-syndecan-1/1:50 dilution for both) at 4°C overnight. Following incubation, the sections were counterstained with hematoxylin, dehydrated, and cleared in xylene. Finally, they were mounted and observed using bright-field microscopy.

A morphological evaluation of the dental mesenchymal tissues at the developing apex was performed in serial sections stained with hematoxylin–eosin. The topographical relationships among the previously described mesenchymal cell populations of the dental pulp, dental papilla, apical papilla, and dental follicle were analyzed.

All sections were evaluated by two experienced observers, KK and AF. Interobserver agreement was assessed using Cohen's *κ* statistic with values >0.8 indicating near-perfect agreement. A *χ*^2^/Fischer's exact test was conducted to compare categorical variables (staining) between dental pulp and apical papilla using the Statistical Package for the Social Sciences, version 24 with the significance level set at 5% (*p* value < 0.05).

## 3. Results

**Figure 2:**
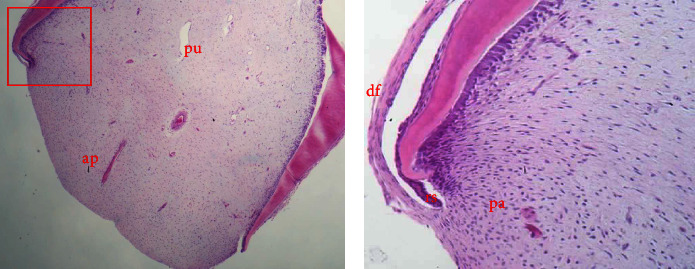
Four distinct anatomical zones of mesenchymal cells can be observed in the area of immature root apex, along the epithelial root sheath (rs). The radicular dental pulp encased within the circumpulpal dentin (pu), the apical papilla (ap) at the most apical portion of the tooth germ connected with the dental follicle (df), and an intermediate cell-rich zone between pulp and apical papilla cells (pa). Original magnification 4x (A) and 40x (B) (*Supporting Information 1*).

Dental mesenchymal cells are clearly distributed into two separated anatomical zones in the developing root apex, the dental pulp and the apical papilla, which is loosely attached to the apex of the tooth (Figure 2).

**Figure 3:**
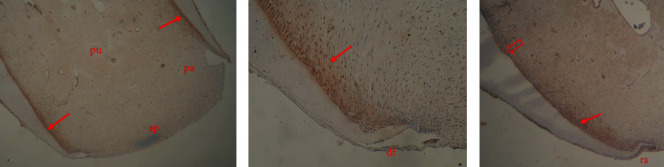
Syndecan-1 expression in the immature root apex of swine premolar. Immunostaining is detected in the epithelial root sheath (rs) and in the odontoblastic layer along the newly deposited root dentin (arrows in A–C). Expression of syndecan-1 cannot be seen along the fully differentiated odontoblasts (arrowhead in C). Apical papilla (ap) and dental follicle as well as the intermediate zone of dental papilla (pa) are negative (A and B). Original magnification 4x (A), 20x (B), and 10x (C).

Syndecan-1 was strongly expressed in the dental pulp, specifically along the root odontoblasts and the newly deposited predentin layer. However, this expression diminished along the fully differentiated odontoblasts located at the coronal pulp (Figure 3). The expression of this cell surface proteoglycan was also observed in the dental pulp surrounding the blood vessels. Notably, there was no syndecan-1 immunostaining detected in the apical papilla and dental follicle cells (*p* < 0.05), as depicted in Figure 3.

**Figure 4:**
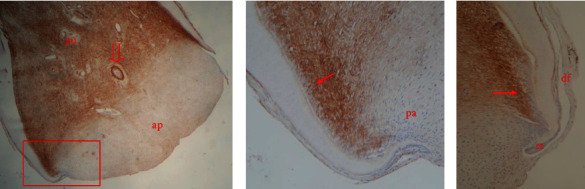
Tenascin-C expression in the immature root apex of swine premolar. Immunostaining is detected throughout the whole pulp tissue (pu). Epithelial root sheath (rs), apical papilla (ap) and dental follicle (df) as well as the intermediate zone of dental papilla (pa) are negative (A and B). More intensive immunostaining is seen in the subodontoblastic cell layers along the newly deposited root dentin (arrows in B and C) and around the pulp capillaries (arrowhead in A). Original magnification 4x (A), 20x (B), and 40x (C).

Tenascin-C was strongly expressed in the entire dental pulp mesenchyme, as shown in Figure 4. Specifically, intense immunostaining for this extracellular matrix protein was observed in the subodontoblastic layer along the newly deposited predentin and surrounding the pulp blood vessels (Figure 3). The apical papilla and dental follicle did not express tenascin-C (*p* < 0.05), as illustrated in Figure 4.

Dental epithelial cells tested negative for both antibodies. However, syndecan-1 was only expressed at the most apical site of the root sheath, as shown in Figures 3 and 4. Detailed results are included in the *Supporting Information 2*.

## 4. Discussion

REPs are considered the treatment of choice for developing teeth with pulp necrosis. The objective is to promote further root development through dental pulp regeneration [28]. Successful outcomes so far are indicated by a thickening of the walls, increase in root length, and narrowing of the root apex [29]. However, both animal studies and human case reports have shown that the thickening and lengthening of dentinal walls or apical closure seen in radiographs should not be attributed to dentin–pulp complex regeneration. Instead, these changes are likely the result of bone-like or cementum-like tissue formation [30–33].

Hard tissue deposition on root canal walls or ingrowth of periodontal-like tissue indicates the absence of differentiated cells committed to the odontoblastic cell lineage, which are necessary for promoting dentin–pulp tissue regeneration. Progenitor cells from blood clots fail to differentiate into odontoblasts, possibly due to the lack of specificity of apical papilla progenitor cells or the presence of progenitors from anatomical zones other than the apical papilla [34]. In a study by Torabinajed et al. [35] it was clearly demonstrated that dental pulp regeneration is successful when preserving the apical 1–4 mm of the dental pulp in immature teeth. Regenerative protocols are challenging in teeth with necrotic pulp because it is difficult to utilize remaining dental pulp cells for the repair process. Additionally, a long-standing root canal infection can decrease the body's ability to regenerate the dentin–pulp complex [36]. According to different tissue engineering strategies, tissue specific cells committed to the odontoblastic cell lineage could be isolated, cultured, and implanted using appropriate scaffolds into the empty root canal space [37, 38]. These studies show that implantation of empty root canals with apical papilla and dental pulp stem cells [37] or dental pulp stem cells only [38] seeded on organic or synthetic scaffolds into the subcutaneous tissue of mice or the postextraction socket of minipigs resulted in the formation of a new layer of dentin-like tissue on the canal dentinal walls. Specifically, the formation of a continuous layer of polarized cells showing typical columnar morphology adjacent to the newly deposited organic matrix was evident. Consequently, tissue specific cells and the appropriate microenvironment are required for the regeneration of the dentin–pulp complex.

In the absence of developmental pathological conditions, peripheral cells of the dental papilla become odontoblasts in a specific temporospatial pattern. Stage-specific enamel epithelium and the intermediate basement membrane play an important role in the initiation of odontoblast differentiation and dentin formation [39–41]. The dental papilla encased within the dentin walls, evolves into pulp tissue, and progressively moves more apically, while the apical papilla remains attached to the apex (Figure 1) [19, 42]. It is widely accepted that the progenitors of apical papilla will form both root pulp tissue and the surrounding periodontium [19]. Moreover, it has been documented that an intermediate papilla mesenchyme called the “apical cell rich zone” exists between the pulp tissue and apical papilla. In our previous study, we reported that this intermediate zone might be considered as the dental papilla providing the source of root odontoblasts [43]. In addition, Sonoyama et al. [6] reported that the apical papilla harbors mesenchymal progenitor cells (SCAP) expressing various mesenchymal stem cell markers that may be the primary source of root odontoblasts. The relationship between cells in the apical papilla and those in the apical cell rich zone should be further investigated. Consequently, the progressive differentiation of apical papilla or apical cell rich zone cells is clearly a process dependant on a timeline schedule defining their regenerative capacity when used for cell homing in regenerative procedures. This illuminates another serious problem that arises when regenerative protocols are applied via cell homing. It is impossible to define whether the cells used originate from the apical papilla, apical cell rich zone, bone marrow, or represent surviving dental pulp cells that seem to undergo a different lineage commitment progression over time.

Thesleff's research team has adequately documented the role of changes in intracellular matrix molecule expression in odontogenic tissue differentiation [23, 24]. Tenascin, also known by other names including hexabrachion, due to its hexabrachion structure, and cytotactin, is a large extracellular glycoprotein expressed in the condensed mesenchyme of many developing organs [25, 44]. Expression of tenascin-C can be detected at the early bud stage in the mesenchyme [25] and in the dental papilla when odontoblasts differentiate [23]. Syndecan-1 is a cell surface heparin sulfate proteoglycan that binds molecules guiding various cellular interactions [45, 46]. During odontogenesis, syndecan-1 immunostaining has been shown to be intense in the dental follicle, in the dental pulp close to the dental follicle, and around the pulpal capillaries [47, 48].

Furthermore, experimental tissue recombination studies have shown that the expression of both molecules in the dental mesenchyme is accompanied by the induction of mesenchymal cells into the dental cell lineage. This fact may be associated with the commitment of these cells to differentiate into odontoblasts [15, 16]. Additionally, it has been demonstrated that tenascin and syndecan are strongly expressed in determined, yet relatively undifferentiated cells, but this expression is lost when these cells undergo terminal differentiation [23].

In the present study, the expression patterns of both tenascin-C and syndecan-1 were associated with the progressive differentiation of odontoblasts, expressed in newly differentiated odontoblasts at the apical part of the pulp but not in the apical papilla mesenchyme. The results showed that the dental pulp exhibited a distinct expression pattern of both molecules, which differed statistically significantly from the apical papilla which remained unstained.

These results are consistent with previously published data [26, 47–50]. Both molecules are present during odontoblast differentiation [26], suggesting that the cells residing in the newly formed apical pulp are committed to the odontoblastic lineage through epithelial–mesenchymal interactions. In contrast, the apical papilla tissue is a tenascin-C and syndecan-1 free zone indicating that specific epithelial–mesenchymal interactions do not occur there. It is likely that cell differentiation potential is progressively restricted to the newly formed dental pulp, and therefore, the phenotypic expression of apical papilla progenitor cells remains undetermined. Consequently, the regeneration of the dentin–pulp complex cannot be achieved using these cells via cell homing unless the new microenvironment induces cell differentiation toward the odontoblastic lineage. However, histological results from studies on regenerative procedures for immature teeth indicate that this is not accomplished, even when inactive scaffolds are used to support cell adhesion and proliferation [51]. These results indicate the importance of localized environmental conditions in the lineage commitment progress of mesenchymal cells residing in the developing apex. This fact should not be ignored when applying tissue-specific regeneration procedures. Though preclinical study results should be cautiously transferred to clinical conditions potential considerations towards more targeted treatment strategies should be involved. Better results can be achieved by improving cell seeding techniques such as cell sheet technology [52] or by enhancing the ability of microenvironments to stimulate the regeneration process through increased numbers and concentrations of growth factors in bioactive scaffolds like platelet-rich plasma (PRP) and platelet-rich fibrin (PRF) [53].

This animal study is the first to provide evidence on the immunohistochemical expression of tenascin-C and syndecan-1 in the developing swine root apex. Both molecules have been proven to be present in differentiating sites of the dental mesenchyme. Their presence in the extracellular matrix and role in cellular interactions indicate a microenvironment that favors odontoblast differentiation. In tissues where these molecules are not expressed, such as the apical papilla, these interactions do not occur, and cellular commitment to odontogenic lineage is lacking. This knowledge may be a prerequisite for understanding the effectiveness of clinical regenerative procedures. Additionally, the experimental model used appears to be suitable, as the pig is considered one of the most appropriate animals for clinical dental research.

The present study is not free from certain limitations. This histomorphometric study was conducted on a porcine animal model and the results should not be directly generalized to humans. Although it has been adequately documented that the expression pattern of both molecules evaluated is not different from that in humans and that the porcine model presents proper analogies and resemblances to human any conclusions should be cautiously interpreted. In addition, a quantitative or semiquantitative evaluation of the expression of these molecules was not performed. Since the apical papilla zone was not stained in any specimen and given the fact that it has been adequately documented by previous published data that these molecules are expressed at the early stage of pulp mesenchymal cell differentiation it could be concluded that the apical papilla zone is a relatively undifferentiated zone where epithelial–mesenchymal interactions are still inactive. Additionally, the determination of the regenerative potential of different mesenchymal cell populations using tenascin-C and syndecan-1 presents another limitation. Other methods, such as the ex vivo evaluation of cell multilineage differentiation and proliferation potential, target protein expression, or *in vivo* evaluation through cell transplantation experiments could be used in combination to provide solid evidence [54].

According to the tenascin-C and syndecan-1 expression patterns presented in this study, the mesenchymal cell differentiation potential in the developing swine apex is progressively restricted to the newly formed dental pulp. The phenotypic expression of apical papilla cells is still undetermined, indicating that the regeneration of the dentin–pulp complex cannot be achieved through cell homing procedures alone. Instead, a new microenvironment must induce cell differentiation to the odontoblastic lineage. *In vivo* characterization procedures can only determine the area specific potential of dental mesenchymal progenitor cells. Emphasizing the importance of well-designed preclinical studies, the *de novo* differentiation of dental progenitors into polarized odontoblasts forming a tubular matrix in a predentin-like pattern remains the appropriate criterion to characterize dentin–pulp regeneration and advance future dental stem cell research efforts and regenerative procedures. Preclinical evaluation in high quality animal studies remains a basic prerequisite indicating the clinical benefits of REPs that will be validated by well-designed clinical trials.

## Data Availability

The data used to support the findings of this study are available from the corresponding author upon request.

## Supporting Information

Supporting Information 1 Original uncropped/unadjusted image for photograph in the Figure 2A. C: original magnification of the Figure 2A. 2B: Original uncropped/unadjusted image for photograph in the Figure 2B.

Supporting Information 2 Results of Syndecan-1 and Tenascin-C expression in the dental pulp and apical papilla cell population by the two observers.
