# Supplementary figures and images for: The Differentiation Potential of Apical Papilla Cells in Relation to Tenascin-C and Syndecan-1 Expression and Their Potential Role in Regeneration
Source: Int J Dent. 2024 Sep 20;2024:7295498. doi: 10.1155/2024/7295498 (PMC11436271; doi:10.1155/2024/7295498)

Figure 2A

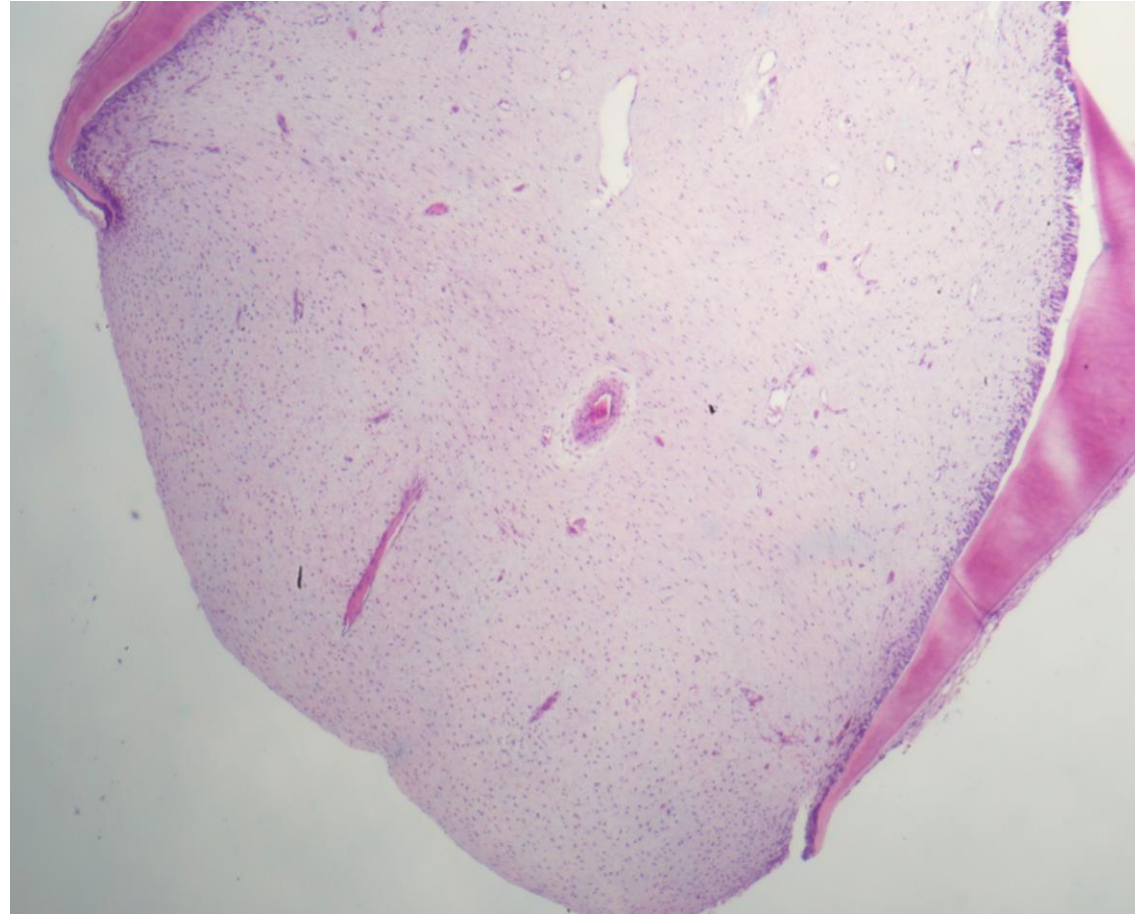

Figure 2B

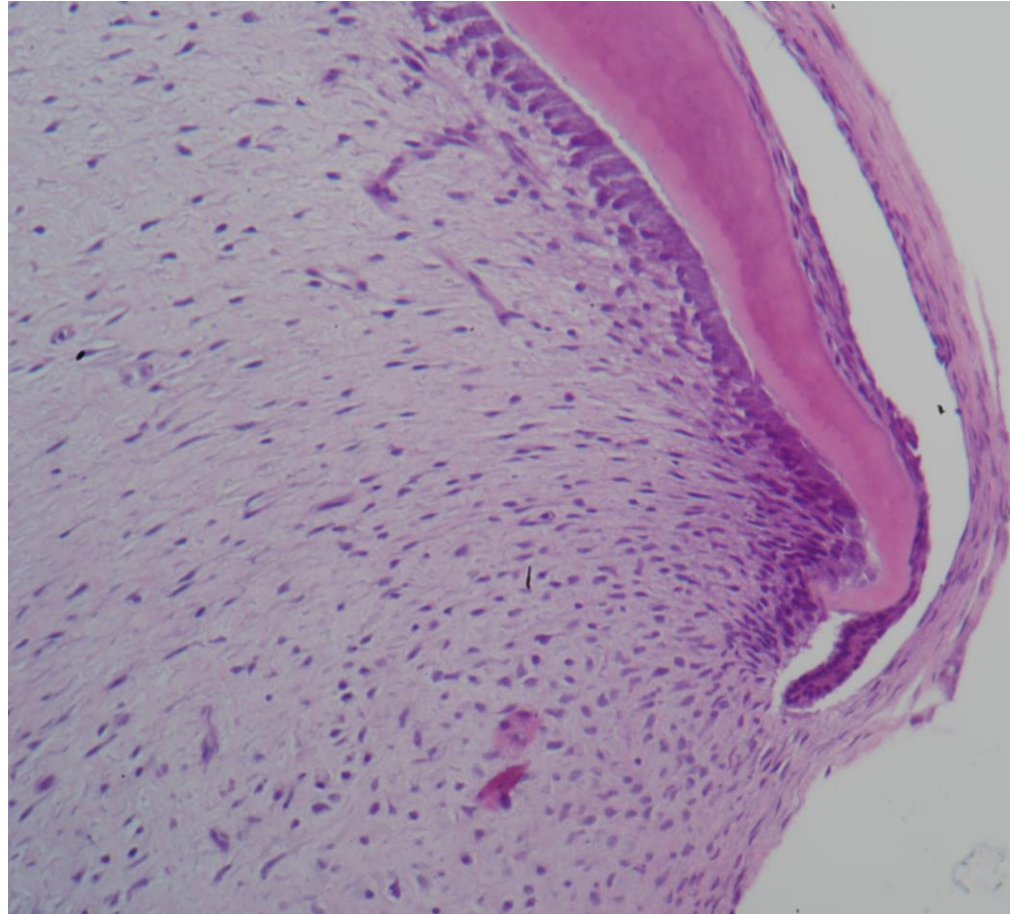

Figure C (detail of 2A)

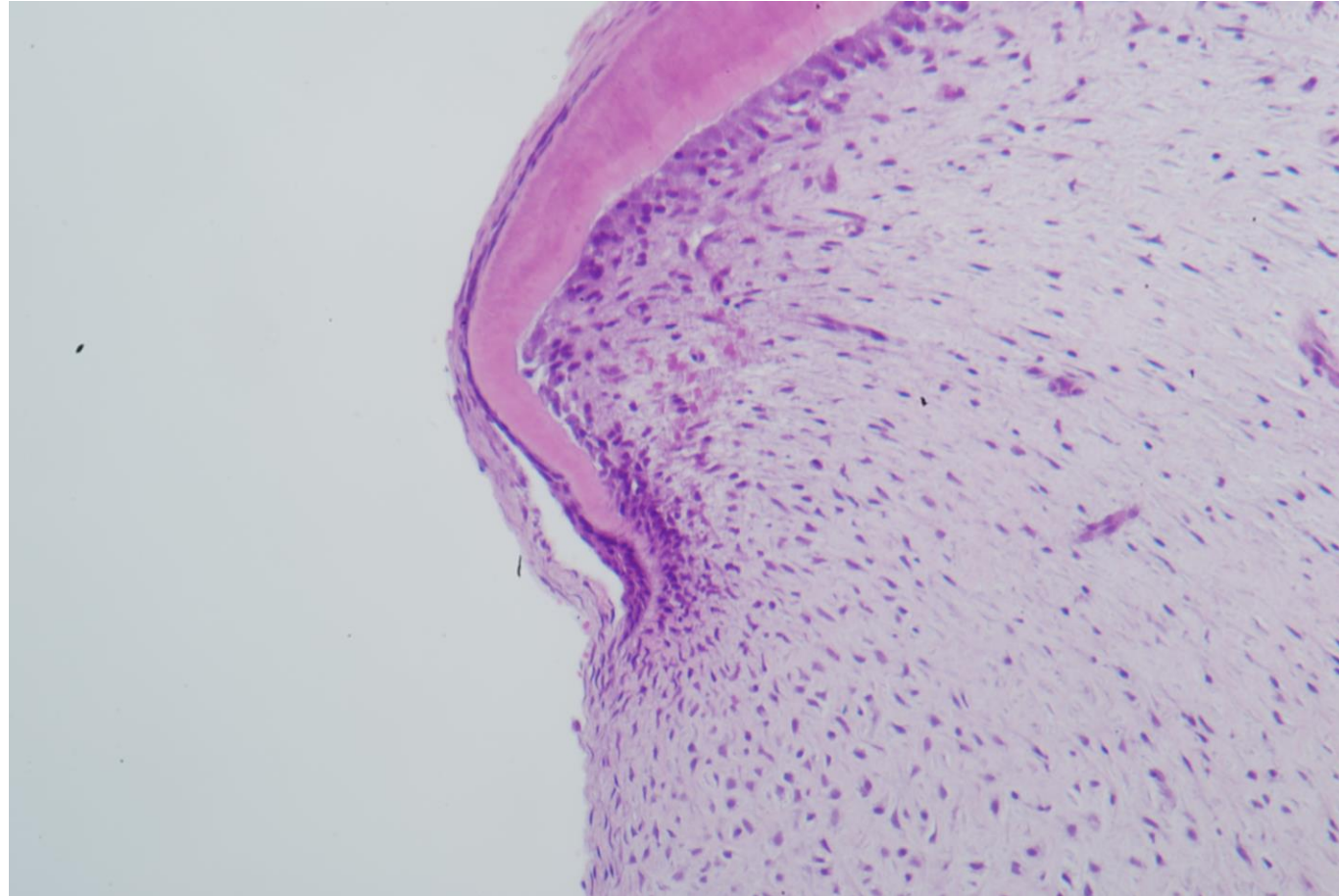

Supplement: Supporting Information 1 — Original uncropped/unadjusted image for photograph in the Figure 2A. C: original magnification of the Figure 2A. 2B: Original uncropped/unadjusted image for photograph in the Figure 2B. [file 7295498.f1.pdf]
