# Supplementary material for: The Differentiation Potential of Apical Papilla Cells in Relation to Tenascin-C and Syndecan-1 Expression and Their Potential Role in Regeneration
Source: Int J Dent. 2024 Sep 20;2024:7295498. doi: 10.1155/2024/7295498 (PMC11436271; doi:10.1155/2024/7295498)
Supplement: Supporting Information 2 — Results of Syndecan-1 and Tenascin-C expression in the dental pulp and apical papilla cell population by the two observers. [file 7295498.f2.docx]

|  |  | **KK** | | | **AF** | | |
| --- | --- | --- | --- | --- | --- | --- | --- |
|  |  | **Syndecan-1** | | | **Tenascin-C** | | |
|  |  | **Dental pulp** | **Apical papilla** | **Dental follicle** | **Dental pulp** | **Apical papilla** | **Dental follicle** |
| **Mini pig 1** | **Incisor 1** | **+** | **̶** | **̶** | **+** | **̶** | **̶** |
|  | **Incisor 2** | **+** | **̶** | **̶** | **+** | **̶** | **̶** |
|  | **Premolar 1** | **+** | **̶** | **̶** | **+** | **̶** | **̶** |
|  | **Premolar 2** | **+** | **̶** | **̶** | **+** | **̶** | **̶** |
| **Mini pig 2** | **Incisor 1** | **+** | **̶** | **̶** | **+** | **̶** | **̶** |
|  | **Incisor 2** | **+** | **̶** | **̶** | **+** | **̶** | **̶** |
|  | **Premolar 1** | **+** | **̶** | **̶** | **+** | **̶** | **̶** |
|  | **Premolar 2** | **+** | **̶** | **̶** | **+** | **̶** | **̶** |
| **Mini pig 3** | **Incisor 1** | **+** | **̶** | **̶** | **+** | **̶** | **̶** |
|  | **Incisor 2** | **+** | **̶** | **̶** | **+** | **̶** | **̶** |
|  | **Premolar 1** | **+** | **̶** | **̶** | **+** | **̶** | **̶** |
|  | **Premolar 2** | **+** | **̶** | **̶** | **+** | **̶** | **̶** |

**Table 1. Results of Syndecan-1 and Tenascin-C expression in the dental pulp and apical papilla cell population by the two observers.**
